# Role of Microbiota in Pathogenesis and Management of Viral Hepatitis

**DOI:** 10.3389/fcimb.2020.00341

**Published:** 2020-08-11

**Authors:** Rashi Sehgal, Onkar Bedi, Nirupma Trehanpati

**Affiliations:** Department of Molecular and Cellular Medicine, Institute of Liver and Biliary Sciences, New Delhi, India

**Keywords:** hepatitis, fecal microbiota transplantation, gut microbiota, lipopolysaccharides (LPS), probiotic, bacteria

## Abstract

Hepatitis is a condition that can be self-limiting or can progress to fibrosis (scarring), cirrhosis, or liver cancer. These days, gut microbiota becomes an important part of our immune system, which is important for disease progression or recovery. Translocation of gut microbial and metabolic products causes intestinal inflammation by modulating immune cells leading to impairment of the primary barrier. But there are limited studies discussing pathogenesis and management of hepatitis with gut microbiota. In this review, we have discussed the role of gut microbiota in pathogenesis and management of various hepatitis, especially hepatitis B and C. We have discussed the role of bacterial products, LPS-TLR4 pathway, and unmethylated CpG DNA, which ultimately affects downstream NF-kB signaling in hepatitis. Finally, we have discussed the role of fecal microbiota transplantation in the management of hepatitis.

## Introduction

Hepatitis is generally known as an inflammation of the liver that can be caused by hepatic and non-hepatic viruses, can be caused by alcohol, can be drug induced, and can be caused by autoimmunity. Gut microbiota composition is known to be associated with disease pathogenesis. However, dynamic alteration of the gut microbiota in disease pathogenesis is not well-understood.

Microbiota involves communities of commensal, symbiotic, as well as pathogenic microorganisms found in organisms, i.e., plants and animals. Microbiota of a healthy individual shows more of commensalism or symbiosis without causing any disease. These microbes mainly colonize humans during birth or shortly thereafter and remain throughout the course of life. These can be found in many areas like skin, respiratory tract, urinary tract, and digestive tract, while brain, lungs, and the circulatory system are free of microbes. Approximately 10^14^ microbes are present in a healthy individual gut (Minemura and Shimizu, 2015). Therefore, gut microbiota has an important role to modulate the immune system in disease progression or recovery.

Translocation of microbes or their metabolic products cause intestinal inflammation leading to impairment of the primary barrier (Hill et al., 2010). There is limited available information regarding the role of gut microbiota in hepatitis, which makes it important to majorly focus on clinical data of gut microbiota linked with hepatitis B and C virus.

## Gut Microbiota

Gut or gastrointestinal tract starts from the mouth and ends at the back passage (anus). Gut helps in the digestion of food by absorbing energy and nutrients. Majority of gut microbiota (80 to 85%) contains good bacteria, and only 15 to 20% are harmful bacteria in different parts of the intestine (Bajaj et al., 2014). In mouth and upper respiratory tract, normal flora is more of **the** commensal bacteria like *Streptococcus, Moraxella, Neisseria*, and *Haemophilus*. Very few species of bacteria are present in the stomach and small intestine, while the large intestine and colon contain dense population of microbes, i.e., up to 10^12^ cells/g. Along with bacteria, many other microorganisms like fungi, protists, archaea, and viruses also symbiotically harbor in the gut.

There are four dominant phyla of bacteria present in the gut, and they are *Firmicutes*, Bacteroidetes, *Actinobacteria*, and Proteobacteria (Khanna and Tosh, 2014). Most important genera in which bacteria belong are *Bacteroides, Clostridium, Faecalibacterium, Eubacterium, Ruminococcus, Peptococcus, Peptostreptococcus*, and *Bifidobacterium* (Fernández et al., 2018). Some of the fungal species that also coexist in the gut are *Candida, Saccharomyces, Aspergillus, Penicillium, Rhodotorula, Trametes, Pleospora, Sclerotinia, Bullera*, and *Galactomyces*, among others (Raimondi et al., 2019).

### Functions of Gut Microbiota

Gut microbiota plays an important but diverse role such as barrier effect, vitamin synthesis, and fermentation. Resident bacteria of the gut acts as a barrier and protect the intestinal mucosa from invasion of the other potential pathogens (Hooper et al., 1999). Many factors including diet, age, medication, illness, stress, and lifestyle influence the gut microbiota, which have a great impact on disease pathogenesis. In fact, many bacteria, i.e., *Bacteroides, Eubacterium, Propionibacterium*, and *Fusobacterium*, are instrumental in the synthesis of vitamins K and B (i.e., folate, B12, and biotin) (Canny and McCormick, 2008). They are also involved in the fermentation of non-digestible carbohydrates for the production of short-chain fatty acids (SCFAs), which are helpful in maintaining metabolic homeostasis. In addition to the production of SCFA, glycolysis and pentose phosphate pathway also produce butyrate, which promotes the growth of *Lactobacilli* and *Bifidobacteria* bacteria in the colon (Venegas et al., 2019). Various studies supported the fact that nutrients derived from microbiota play a pivotal role in the normal functioning of the hepatic system (Li et al., 2012; Zheng et al., 2013; Moratalla et al., 2014; Jiminez et al., 2016; Cremer et al., 2017; Wang et al., 2017a).

### Gut Microbiota in Liver Diseases

**Figure 1:**
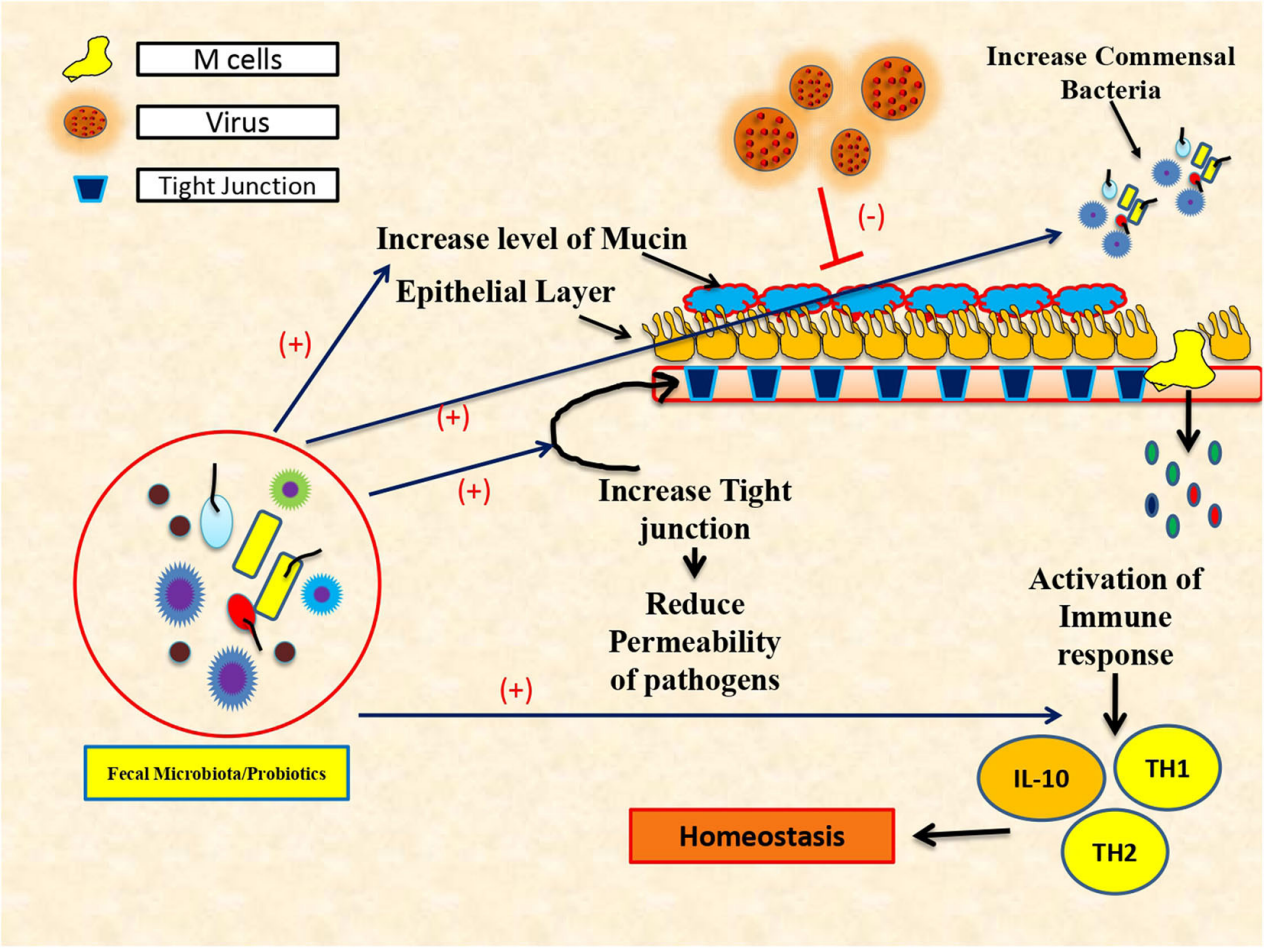
Protective role of fecal microbiota transplantation and use of probiotics in immune restoration.

Commensal bacteria play a decisive role in maintaining immune homeostasis (Figure 1) and also guard immune reactions at mucosal surfaces (Ichinohe et al., 2011). Intestinal microflora is a dynamic and complex ecosystem, which helps in proliferation, growth, and differentiation of epithelial cells to fight infections and improve immunity. Despite its crucial role in the synthesis of vitamin K, folate, SCFA, and peroxides, gut microbiota acts as a chief environmental as well as etiological factor for the progression of many liver diseases (O'Hara and Shanahan, 2007). Particularly, gut microbiota has a larger influence on alcoholic liver disease, non-alcoholic fatty liver disease, viral hepatitis (hepatitis B and C), autoimmune hepatitis (AIH), primary sclerosing cholangitis (PSC), and primary biliary cholangitis (PBC) (Mohamadkhani, 2018). *Lactobacillus, Bifidobacterium, Saccharomyces boulardii*, and *Lactobacillus plantarum* play a bigger role in the management of various metabolic disorders and hepatitis (Mohamadkhani, 2018).

Several pathogens, including viruses and intestinal microorganisms, use mucous membranes as a doorway (Karst, 2016). Hepatic viruses breach the intestinal permeability leading to gut dysbiosis and release pro-inflammatory cytokines instrumental in developing liver cirrhosis and HCC. It is also observed that the use of probiotics reduces the tolerogenic response and enhances the mucosal defense against viral pathogens (Rigo-Adrover M del et al., 2018). *Lactobacillus* alone can influence the production of interferon by modulating the antiviral effects of vitamin A (Lee and Ko, 2016). The mixture of various probiotics and *Bifidobacterium* with galacto-oligosaccharides and fructo-oligosaccharides has a defensive effect against *Rotavirus* infection by aggregating the production of TNF-α, IL-4, IFN-γ, and TLR2 expression (Rigo-Adrover M del et al., 2018). In most of the liver disease, especially cirrhosis, dysbiosis of the gut increases Proteobacteria, *Enterobacteriaceae*, and *Veillonellaceae*, while it decreases Bacteroidetes and *Lachnospiraceae* (Sanduzzi Zamparelli et al., 2017). Recently, the cirrhosis dysbiosis ratio (CDR) is coined for defining the changes in gut microbiome in cirrhosis patients with beneficial *Lachnospiraceae* and *Ruminococcaceae* and harmful *Enterobacteriaceae* bacteria (Bajaj et al., 2014). Other groups have also associated patients with severe cirrhosis and hepatic encephalopathy with overgrowth of *Enterobacteriaceae* bacteria (Chen et al., 2011).

## Role of Gut Microbiota in Hepatic Viral Infections

Acute viral hepatitis due to hepatitis A and E viral infections is a major community health problem especially in developing countries. Hepatitis A and E cause acute infection which could be short-lived and self-clearing unless the subjects are immunocompromised or in transplant settings. Acute hepatitis E infection also becomes detrimental and life-threatening during pregnancy, affecting both the mother and the child.

Both hepatitis A and E are RNA viruses that transmit through oral fecal routes (Lemon et al., 2018) and may have devastating effects on intestinal microflora. It was observed that administration of the healthy probiotic bacterium like *Enterococcus faecium* NCIMB 10415 affects the reduction as well as the removal of enteric HEV viruses in pigs (Kreuzer et al., 2012). However, there is lack of relevant data in humans.

As per the World Health Organization (WHO), hepatitis B virus (HBV) infection caused 887,000 deaths in 2015 and 4.5 million (16.7%) diagnosed with chronic infection in 2016. Similarly, hepatitis C virus (HCV) caused 399,000 deaths with an estimated 71 million diagnosed with chronic infection in 2016. Both these viruses cause chronic infections at 10% in HBV and more than 30% in HCV leading to cirrhosis and hepatocellular carcinoma. Hepatic viruses have evolved mechanisms to avoid their detection from the host innate and adaptive immunity and characterized as viral escape (Visvanathan et al., 2007). It is observed that chronic hepatitis patients have larger translocation of the intestinal microbiota (Lu et al., 2011; Li et al., 2018).

Bacterial translocation cause intestinal inflammation via dysregulation of immune cell, overgrowth of pathogenic bacteria, as well as dysfunction of the primary barrier (Hill et al., 2010). Xu et al. (2015) also supported the fact that intestinal flora loses homeostasis during dysbiosis, which in fact helps the advancement of hepatitis viral infection (Xu et al., 2015).

Therefore, it is now understood that during chronicity, commensal microbiota have greater impact not only on viral host cell interaction but also on viral replication.

In viral hepatitis, few harmful bacteria like *Escherichia coli, Enterobacteriaceae, Enterococcus faecalis, and Faecalibacterium prausnitzii* directly alter the profile of good intestinal microbiota with a lower number of intestinal lactic acid species such as *Lactobacillus, Pediococcus, Weissella*, and *Leuconostoc* (Bajaj et al., 2014; Chen et al., 2016). Some of the bacterial species, i.e., *Neisseria, E. coli, Enterobacteriaceae, E. faecalis, F. prausnitzii*, and *Gemella*, are also found responsible for the progression of hepatitis B and C virus–related cirrhosis and primary biliary cirrhosis (Chen et al., 2016; Mohamadkhani, 2018). *Candida* is also frequently found in patients with hepatitis B–related cirrhosis (Cui et al., 2013).

### Role of Gut Microbiota in Hepatitis B Viral Infection

Dysbiosis of gut microbiota in chronic hepatitis B infection affects disease pathogenesis and causes liver failure in a large proportion. LPS (lipopolysaccharides) from the outer membrane of gram-negative bacteria help in the activation of innate immune response by recognizing TLRs, especially TLR2 and 4. HBV infection leads to progressive decline in butyrate-producing bacteria. However, LPS-producing genera is enriched in HBV infection. In HBV infection, a beneficial bacterium, *Lachnospiraceae*, plays a role in the management of HBV infection via reduction in LPS section and bacterial translocation (Chen et al., 2011; Ren et al., 2019). Studies have shown the role of Faecalibacterium*, Pseudobutyrivibrio, Lachnoclostridium, Ruminoclostridium, Prevotella, Alloprevotella*, and *Phascolarctobacterium* in potential anti-inflammatory SCFA activity, which increases the abundance of butyrate compared to normal subjects (Liu et al., 2019). Lu et al. (2011) have demonstrated that copy numbers of *F. prausnitzii, E. faecalis, Enterobacteriaceae, Bifidobacteria*, and lactic acid bacteria (*Lactobacillus, Pediococcus, Leuconostoc*, and *Weissella*) have marked variation in the intestine of HBV cirrhotic patients. During HBV infection, dysbiosis in the oral microbiota was observed, and yellow tongue coating is suggestive of a reduction in Bacteroidetes but an increase in Proteobacteria. Zhao et al. (2018) also suggested positive correlation of *Neisseriaceae* with the serum HBV-DNA.

Cirrhotic patients with HBV infection showed a significant decrease in the *Bifidobacteriaceae*/*Enterobacteriaceae* (B/E) ratio (Lu et al., 2011), while Yun et al. observed no difference in the B/E ratio in HBsAg+ with normal or high ALT and in non-cirrhotic HBV carriers (Yun et al., 2019). It means the B/E ratio is disturbed only in cirrhosis. However, other study observed that the *Megasphaera* genus from the Firmicutes phylum was abundant in the HBsAg+ high ALT group than the normal ALT. In patients with normal ALT, butyrate-producing bacteria like *Anaerostipes* are more in feces compared to HBsAg-ve (Yun et al., 2019). It is interesting to note that both *Megasphaera* and *Anaerostipes* produce SCFA as a by-product of lactate fermentation and butyrate. However, butyrate is known as anticarcinogenic and anti-inflammatory, and plays a role in oxidative stress (Hamer et al., 2008). Another study suggests that chronic hepatitis B infected cirrhotic patients exhibit a decrease in *Bifidobacteria* and *Lactobacillus* levels, while significantly increasing *Enterococcus* and *Enterobacteriaceae* levels compared to healthy individuals.

Bacterial translocation is also observed in the development of hepatocellular carcinoma (HCC). Recently, Wang et al. have defined the serum zonulin as an intestinal permeability marker and showed its association with AFP levels in HBV-associated liver cirrhosis and HCC. They are helpful in correlating it with advanced stages of the diseases (Fasano, 2012).

The use of probiotic in HBV-infected patients showed benefit and suggested that probiotic VSL#3 plays an important role in the management of HBV viral infection (Dhiman et al., 2014).

### Role of Gut Microbiota in Hepatitis C Viral Infection

Chronic hepatitis C infection is another leading cause of cirrhosis, HCC, and in some cases, liver failure and death. In majority, *Enterobacteriaceae* and *Bacterioidetes* increased in chronic HCV patients, but *Firmicutes* found to be decreased. HCV infection cause marked elevation in LPS, which is suggestive of microbial translocation and inflammation during disease progression (Dolganiuc et al., 2007; Inoue et al., 2018). On the other hand, it was observed that antiviral treatment of HCV with ribavirin (RBV) and immune modulator pegylated interferon (PEG-IFN) has no direct impact on gut dysbiosis. In fact, it increases the production of bile acids, which is important for gut microbiota (Ponziani et al., 2018). Some pathogenic bacteria such as *Enterobacteriaceae, Staphylococcus*, and *Enterococcus* decreased the bile acid in HCV-infected cirrhotic patients, which normalized after a direct-acting antiviral treatment. Oral direct-acting antivirals (DAAs) were also found to be helpful in improving gut especially *Lachnospira* and *Dorea* genera, and restored TNFα levels (Pérez-Matute et al., 2019,?).

But after DAA treatment, expression of calprotectin, ZO1, and LPS was found more in HCV patients with cirrhosis. It was also suggested that during HCV infection, *L. acidophilus* and *Bifidobacterium* spp. can act as a supportive supplement with antiviral and antibacterial activities (Dore et al., 2014). Immune response in HCV patients can be stimulated by useful microbiota via activation of CD3+ cells and CD56+ NK cell counts, which were explained by Doskali et al. (2011) and further suggested that good flora increases the cytotoxic effects of NK cells against viral infected cells inhibiting the replication of HCV. Use of probiotics in HCV-infected patients with cirrhosis was significantly beneficial (Preveden et al., 2017).

Another hepatic virus, hepatitis D virus, is a new player and not much is known about it yet. It was also suggested that endotoxemia in HCV and HDV patients seems to be multifactorial, likely depending on impaired phagocytic functions and reduced T-cell-mediated antibacterial activity (Kefalakes and Rehermann, 2019).

## Microbiota Modulates Molecular Signaling in Hepatitis

LPS is the key component of gram-negative bacteria, i.e., *Enterobacteriaceae*. The active receptor for LPS is CD14/TLR4/MD2 receptor complex on induction, which secretes many pro-inflammatory cytokines including tumor necrosis factor-α, IL-1, IL-6, and chemokines through the NF-κB signaling (Fooladi et al., 2010; Seki and Schnabl, 2012; Bryant et al., 2015) to cause liver injury. In the intestinal tract, LPS downregulates the expression of various tight junction proteins (ZO-1 and closed protein) by increasing the permeability of the intestinal mucosa and enters the blood flow through the portal venous system (Park et al., 2010). In liver, Kupffer cells as specialized macrophages are induced by the LPS-TLR4 pathway for the release of immunosuppressive mediators, such as IL-10, which in turn suppress the release of inflammatory mediators by Kupffer cells (Dixon et al., 2013). In this way, during viral hepatitis, virus specific immune responses are suppressed and ultimately inhibit efficient clearing of bacteria as well as viruses.

In addition to LPS, unmethylated CpG DNA, bacterial DNA/RNA bacterial cell wall also contains teichoic acid, peptidoglycan, and specialized proteins (flagellin). Bacterial DNA/RNA is recognized by TLRs as well as all components of cell-wall-like teichoic acid and peptidoglycan also recognized by TLR2, while TLR5 got activated by flagellin. dsRNA bacteria are recognized by TLR3. ssRNA activates receptors of both TLR7 and TLR8. All these TLRs ultimately stimulate the JAK-STAT pathway. Hepatitis viruses are also recognized by TLRs in the liver or in the intestine and activate downstream signaling pathways (Mencin et al., 2009).

Unmethylated CpG DNAs are found abundantly in the *Lactobacillus* family, i.e., *L. casei, L. plantarum, L. rhamnosus*, and others like *Bifidobacteria*, Proteobacteria, and Bacteroidetes in the intestinal flora of animals. Unmethylated CpG DNA is sensed by TLR9, expressed on various mononuclear cells, and stimulates both innate immune response as well as adaptive immune response (Krieg, 2006; Kauppila et al., 2013). Activation the of CpG-TLR9 pathway stimulates downstream molecules of MyD88 such as IRAK4, TRAF6, and IRAK1, ultimately triggering NF-κB and MAPK signaling pathways. These downstream pathways help in the activation of DCs for the secretion of cytokines and chemokines (Krieg, 2006; Kauppila et al., 2013). Chronic HBV patients have reduced *Lactobacillus* and *Bifidobacteria*. Both are rich in unmethylated CpG DNA levels, ultimately affecting the CpG DNA-TLR9 pathway and immune response on HBV (Lin and Zhang, 2017).

## Role of Fecal Microbial Transplantation (FMT) in Viral Hepatitis

FMT mainly involves the insertion of healthy microbiota in the diseased gut. In brief, fecal matter derived from a healthy family member of the patient receiving the same diet as the patient is processed and introduced in the intestinal tract of the patient. These have minimal side effects and proved helpful in reinstating healthy gut flora in the patient. FMT administration can be done using several routes such as oral, nasogastric, nasoduodenal, nasojejunal, endoscopic, rectal, and colonoscopic or midgut transendoscopic enteral tubing (Cui et al., 2015; Tang et al., 2017). For cirrhotic patients with dysbiosis, small bowel route is most affected, while mostly used route is oral delivery. In severe alcoholic hepatitis (SAH), in comparison to steroids, FMT is associated with decreased disease severity and improved survival. Earlier, Wang et al. (2017b) have observed that FMT restored the cognitive function, liver function indexes, and TLR response in carbon tetrachloride (CCl_4_)-induced acute hepatitis in rats.

Woodhouse et al. have observed in a PROFIT clinical trial the benefits of fecal microbiota transplantation in the small bowel of cirrhotic patients (Woodhouse et al., 2019). Meiglani et al. (2020) also observed that cirrhotic patients with antibiotic-resistant *Clostridioides difficile* infection (CDI) responded well after FMT treatment. In fact, fecal microbiota of alcohol-resistant mice when given to alcohol-sensitive mice has reduced Bacteroidetes and increased *Actinobacteria* as well as *Firmicutes* and protected steatosis development (Ferrere et al., 2017). Limited studies are published yet on FMT administration in alcohol-related liver disease. However, all these studies showed immense benefit of FMT. Bajaj et al. (2017) observed the recovery of cognitive function and hepatic encephalopathy in patients under clinical trial after administration of FMT. Studies recently published from our center have found better efficiency of FMT in severe alcoholic patients than standard medical treatment (Sarin et al., 2019). There are only a couple of randomized FMT clinical trials for chronic hepatitis B infected patients (Table 1).

**Table 1:** Randomized FMT clinical trials for the treatment of chronic hepatitis B infection.

**S.No**	**Study title**	**Study type**	**No. of subjects**	**Intervention/Treatment**	**Status**	**Phase**	**Primary outcome measures**	**Secondary outcome measures**	**ClinicalTrials.gov Identifier:**
1.	Randomized Controlled Trial Comparing the Efficacy and Safety of FMT in Hepatitis B Reactivation Leads to Acute on Chronic Liver Failure. **Location:** Institute of liver and Biliary Sciences New Delhi, Delhi, India	Interventional (Clinical Trial)	64	Drug: Tenofovir Drug: Fecal Microbiota Transplantation (FMT)	Completed	Completed	Transplant free survival. [Time Frame: 3 months]	Reduction in Hepatitis B Virus DNA level ≥ 2 log. [Time Frame: 2 weeks] Improvement in MELD (Model for End Stage Liver Disease) score. [Time Frame: 2 weeks]	NCT02689245
2.	Study on Effect of Intestinal Microbiota Transplantation in Chronic Hepatitis B **Location:** Zhongshan Hospital Affiliated to Xiamen University Xiamen, Fujian, China	Interventional (Clinical Trial)	60	Other: intestinal microbiota transplant Drug: Antiviral Agents	Recruiting	N.A.	Change of serum hepatitis B virus e antigen(HBeAg) level [Time Frame: 1, 3, 6 months] Serum hepatitis B virus e antigen(HBeAg) levels is measured in S/CO	Change of serum hepatitis B virus surface antigen(HBsAg) level [Time Frame: 1, 3, 6 months] Serum hepatitis B virus surface antigen(HBsAg) levels is measured in IU/mL. Change of serum anti-hepatitis B virus e antigen(anti-HBe) [Time Frame: 1, 3, 6 months] Appearance of serum anti-hepatitis B virus e antigen(anti-HBe) suggest the ability of body to resistant HBV. Change of serum anti-hepatitis B virus surface antigen(anti-HBs) [Time Frame: 1, 3, 6 months] Appearance of serum anti-hepatitis B virus surface antigen(anti-HBs) suggest the ability of body to resistant HBV. Changes of gut **microbiota** [Time Frame: 1, 3, 6 months] Alpha and Beta diversity of GI **microbiota** by High-throughput sequencing (16S rRNA) on baseline line and 1, 3, 6 months after treatment Relief of constipation [Time Frame: 1, 3, 6 months]; Relief of diarrhea [Time Frame: 1, 3, 6 months]; Relief of abdominal pain [Time Frame: 1, 3, 6 months] The onset and duration of constipation will be assessed by “Evaluation Score Table of Gastrointestinal Symptoms.”	NCT03429439

Recently, groups have addressed how FMT is modulating immunity in gut and liver. Mucosa-associated invariant T (MAIT) cells are found abundant in liver (20% to 50% of intrahepatic T cells), gut, peripheral blood, as well as lungs. Gao et al. have observed that functional MAIT cells were altered in SAH resulting in more bacterial infection in patients. Alteration in circulating MAIT cells is observed with defective antibacterial cytokine/cytotoxic response against the infection (Gao et al., 2018). They believe that FMT administration has a profound effect on the expression of MAIT cells in alcohol-related diseases.

## Summary and Conclusion

Gut microbiota has an important role in viral, alcoholic, and metabolic liver diseases. Gut microbiota plays a crucial role in modulating the toll-like receptors, NF-κB signaling, janus kinase/signal transducer and transcription (JAK/STAT) pathway, and CD4+T cell activation. Numerous useful microbiotas like *Ruminoclostridium, Faecalibacterium, Lachnoclostridium, Prevotella, Alloprevotella, Pseudobutyrivibrio, and Phascolarctobacterium* play an important role in potentiating anti-inflammatory short chain fatty acid (SCFA) activity and increased the butyrate abundance, which play a crucial role in the management of various hepatitis-related viral infections. Fecal microbiota transplantation became an attractive and safest mode of treatment for the management of various liver diseases especially in severe alcoholic hepatitis. Despite recent publications, there are still gaps in understanding the role of microbiota in viral hepatitis especially in acute HAV and HEV viral infections. Therefore, there is a need to explore more in these infections.

## Author Contributions

RS and OB written the review article. NT provide valuable suggestions, corrected, and revised. All authors contributed to the article and approved the submitted version.

## Conflict of Interest

The authors declare that the research was conducted in the absence of any commercial or financial relationships that could be construed as a potential conflict of interest.
